# Mutations Selectively Evolving Peroxidase Activity Among Alternative Catalytic Functions of Human Glutathione Transferase P1-1

**DOI:** 10.3390/antiox13111347

**Published:** 2024-11-02

**Authors:** Aram Ismail, Bengt Mannervik

**Affiliations:** 1 Department of Biochemistry and Biophysics, Arrhenius Laboratories, Stockholm University, SE-10691 Stockholm, Sweden; aram.ismail@dbb.su.se; 2 Department of Chemistry, Scripps Research, La Jolla, CA 92037, USA

**Keywords:** glutathione transferases, GST P1-1, cumene hydroperoxide, mutant libraries, peroxidase activity, alternative substrates

## Abstract

Glutathione transferases are detoxication enzymes with broad catalytic diversity, and small alterations to the protein’s primary structure can have considerable effects on the enzyme’s substrate selectivity profile. We demonstrate that two point mutations in glutathione transferase P1-1 suffice to generate 20-fold enhanced non-selenium-dependent peroxidase activity indicating a facile evolutionary trajectory. Designed mutant libraries of the enzyme were screened for catalytic activities with alternative substrates representing four divergent chemistries. The chemical reactions comprised aromatic substitution, Michael addition, thiocarbamoylation, and hydroperoxide reduction. Two mutants, R1 (Y109H) and an R1-based mutant V2 (Q40M-E41Q-A46S-Y109H-V200L), were discovered with 16.3- and 30-foldincreased peroxidase activity with cumene hydroperoxide (CuOOH) compared to the wildtype enzyme, respectively. The basis of the improved peroxidase activity of the mutant V2 was elucidated by constructing double-point mutants. The mutants V501 (Q40M-Y109H) and V503 (E41Q-Y109H) were found to have 20- and 21-fold improvements in peroxidase activity relative to the wildtype enzyme, respectively. The steady-state kinetic profiles of mutants R1 and V2 in the reduction of CuOOH were compared to the wildtype parameters. The k_cat_ values for R1 and V2 were 34- and 57-fold higher, respectively, than that of the wildtype enzyme, whereas the mutant K_m_ values were increased approximately 3-fold. A 10-fold increased catalytic efficiency (k_cat_/K_m_) in CuOOH reduction is accomplished by the Tyr109His point mutation in R1. The 23-fold increase of the efficiency obtained in V2 was caused by adding further mutations primarily enhancing k_cat_. In all mutants with elevated peroxidase activity, His109 played a pivotal role.

## 1. Introduction

Aerobic organisms are exposed to reactive oxygen species and electrophilic toxicants emerging intracellularly as well as from external sources. Among numerous protective agents, cellular glutathione (GSH) plays a major role in both animals and plants [1,2]. Glutathione peroxidases catalyze the reduction of peroxides including H_2_O_2_ and organic hydroperoxides using GSH as the reductant [3].

Glutathione transferases (GSTs) are an important class of phase II detoxication enzymes partaking both in cellular defense and in other intracellular processes [4,5,6]. These enzymes catalyze the conjugation of glutathione with endogenous and exogenous electrophilic substrates and promote the elimination of the harmful compounds by making them more water-soluble. Furthermore, some GSTs have evolved to serve as glutathione peroxidases acting primarily on organic hydroperoxides [7].

The family of cytosolic GSTs in humans comprises seven structural classes and multiple different isoenzymes, generated by divergent evolution. The enzymes possess broad catalytic diversity [8], but each GST features its own substrate-selectivity profile [4]. The sequence identity of GSTs belonging to different classes are less than 30%, and usually above 70% within the class. Furthermore, cytosolic GSTs are dimeric proteins with a subunit molecular mass between 20 and 25 kDa and contain one active site per monomer. The active site can further be divided into two binding pockets, one for the binding of the cofactor GSH (G-site), and the other for the binding of the hydrophobic electrophilic substrate (H-site). The structural differences noted in the active site across GST classes, along with varying specific interactions between active site residues and different substrates, contribute to varied substrate specificities [9].

Many studies involving the molecular redesign of GSTs have shown the possibility to enhance or shift the substrate selectivity when there is at least an existing small activity initially. Thus, the enzymes can readily evolve for altered functions. For example, point mutations of the H-site residue Tyr109 into Phe [10] or Val [11] in the human pi-class enzyme cause a change in activity with certain substrates while activity with others is unaffected. A tau-class GST from wheat showed improved catalytic activity towards multiple alternative substrates upon alteration of a few evolutionarily variable residues outside the active site [12]. DNA shuffling mutagenesis on Mu-class enzymes has shown that second generation variants, based on promiscuous low activity first generation variants, can evolve into a high activity variant [13]. Plenty of other work related and unrelated to GSTs have also demonstrated the possibility to alter substrate selectivity [14,15,16,17,18,19,20,21]. Overall, these studies suggest that small changes in structure can give rise to large changes in the activity profile of these enzymes.

We have recently investigated mutant libraries of wildtype human GST P1-1 to find enzymes with enhanced activity with the anticancer prodrug Telcyta [22]. We found that a single-point mutation, Y109H, improved the activity 2.9-fold. Because of the promiscuous nature of GSTs, altering the activity in one dimension may affect the activity in other dimensions of substrate-activity space. Here we explore the activity profiles of our mutant libraries by screening them with four alternative substrates undergoing diverse chemical transformations and identify variant enzymes with greatly enhanced glutathione peroxidase activity. The results illustrate the functional plasticity of the GST protein fold, which has successfully been developed in the natural evolution of diverse functions in both animals and plants [23,24]; furthermore, the results indicate its potential for medical and other biotechnical applications.

## 2. Materials and Methods

### 2.1. Materials

1-Chloro-2,4-dinitrobenzene (CDNB), phenethyl isothiocyanate (PEITC), cumene hydroperoxide (CuOOH), ethacrynic acid (EA), NADPH, glutathione (GSH), glutathione reductase (baker’s yeast), and bovine serum albumin (BSA) together with other chemicals were obtained from Sigma-Aldrich (Darmstadt, Germany).

### 2.2. Expression and Purification of Enzymes

Coding sequences of all GST P1-1 gene (allele *A) variants were optimized by ATUM (Newark, CA, USA) for high-level expression in *Escherichia coli* [22]. The genes were synthesized to express an N-terminal His_6_-tag to enable efficient Ni-IMAC purification and were inserted into the expression vector pD444-NH (T5-His-ORF, Ecoli-Elec D). All mutants were expressed and purified according to a published protocol [15].

### 2.3. Activity Measurements with Alternative Substrates

**Figure 1:**
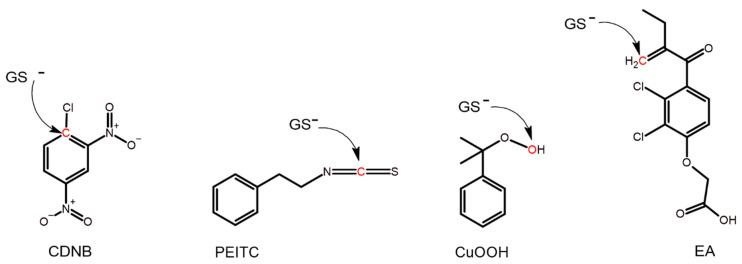
The structure of the alternative substrates used. Atoms marked in red on CDNB, PEITC, CuOOH, and EA are subject to nucleophilic attack by the sulfur atom of glutathione in its reactive thiolate form (GS^−^).

Enzyme activities were monitored spectrophotometrically on a Multiskan GO (Thermo Scientific, Uppsala, Sweden) spectrophotometer. Measurements were performed under standard conditions [25,26] in a 1 mL quartz cuvette at 30 °C, and the structure of each substrate is shown in Figure 1. The following conditions and final concentrations in the cuvette were used for the different substrates: 1 mM CDNB (dissolved in EtOH, 5% in cuvette) with 1 mM GSH in 0.1 M NaH_2_PO_4_/Na_2_HPO_4_ buffer pH 6.5 supplemented with 1 mM EDTA and 0.1% (*w*/*v*) bovine serum albumin (BSA); 0.4 mM PEITC (dissolved in acetonitrile, 2% in cuvette) with 1 mM GSH in 0.1 M NaH_2_PO_4_/Na_2_HPO_4_ buffer pH 6.5 supplemented with 1 mM EDTA and 0.1% (*w*/*v*) BSA; 0.2 mM EA (dissolved in acetonitrile, 2% in cuvette) with 1 mM GSH in 0.1 M NaH_2_PO_4_/Na_2_HPO_4_ buffer pH 6.5 supplemented with 1 mM EDTA and 0.1% (*w*/*v*) BSA; 1.5 mM CuOOH (dissolved in acetonitrile, 1.5% in cuvette) with 1 mM GSH, 0.1 mM NADPH, and 0.3 unit glutathione reductase in 0.1 M NaH_2_PO_4_/Na_2_HPO_4_ buffer pH 7.0 supplemented with 1 mM EDTA and 0.1% (*w*/*v*) BSA. Extinction coefficients: Δε_340_ = 9600 M^−1^ cm^−1^ for CDNB; Δε_274_ = 8890 M^−1^ cm^−1^ for PEITC; Δε_340_ = −6200 M^−1^ cm^−1^ for NADPH; Δε_270_ = 5000 M^−1^ cm^−1^ for EA.

### 2.4. Saturation Curves for Steady-State Kinetic Parameters

The substrate CuOOH was used to carry out saturation experiments to establish steady-state kinetic parameters under regular conditions (see above). The concentrations of CuOOH ranged from 0.09 to 3 mM, while the concentration of GSH was fixed at 1 mM.

### 2.5. Statistical Data Analysis

The software package XLSTAT (version 2024.3.0) was used for multivariate data analysis of mutant enzymes with measurable activity with the alternative substrates. Principal component analysis (PCA) was performed to investigate the relationship between the mutant enzymes and the activities with the selected substrates.

## 3. Results

### 3.1. Mutant Libraries

Enzymes were obtained via bacterial expression and their purity was verified by SDS-PAGE [22]. Recombinant mutant libraries (RX and R2XX, Table 1) were based on rational design targeting both active site residues and residues belonging to the flexible α2-loop (residues 35–46) [27,28] located at the N-terminal portion of the protein.

The first-generation RX mutant library comprised three single-point mutants (Table 1), and the second generation R2XX mutant library contained thirteen double-point mutants (Table 1). Other recombinant mutant libraries (VX, V20X, and V40X, Table 2) were built based on the Y109H mutant and guided by a machine-learning approach using design of experiments [29].

This method utilizes phylogenetic information derived from protein sequence databases. In short, a protein BLAST of human GST P1-1 was performed to acquire homologous sequences. The top 100 non-redundant sequences with >70% sequence similarity were selected for a sequence alignment, and all sites with evolutionarily non-conserved residues and all the naturally occurring amino acid variability within each position were identified. These residues were then scored and ranked based on a set of preselected parameters, for example, Dayhoff PAM matrix for GST P1-1, sequence conservation, and location in protein structure [12,29,30]. The fourteen highest-ranked substitutions were systematically incorporated to generate a total of seventeen gene variants (Table 2). The V3XX mutant library (Table 3) was based on the V20X library (Table 2), with the addition of five new substitutions at three different positions. To the three selected positions substitutions were added either as single-point mutations, or as combinations of double-point mutations and triple-point mutations to construct seventeen mutants (Table 3). Lastly, additional mutants were constructed to establish the individual contributions of each substitution to the catalytic activity of relevant multi-substituted mutants (V50X, Table 2). The mutants belonging to the V3XX and V50X library were successfully expressed and purified

**Figure 2:**
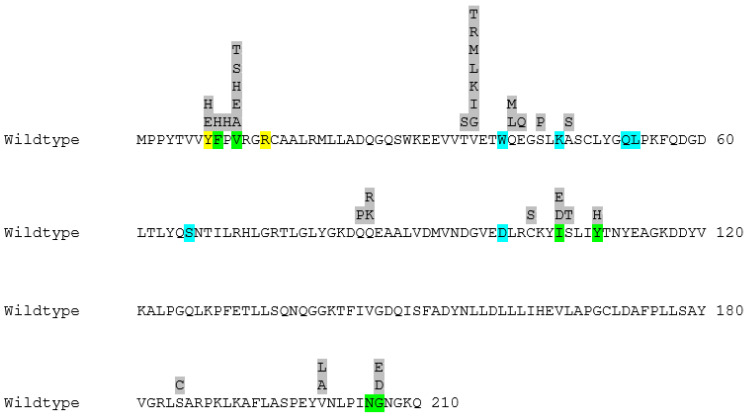
The primary structure of wildtype human GST P1-1 with all the substitutions selected for each position highlighted in grey. The active site of the enzyme can be divided into two binding cavities. Residues highlighted in blue belong to the cavity responsible for binding GSH (G-site), whereas residues highlighted in green belong to the cavity responsible for binding the electrophilic substrate (H-site). The two residues showcased in yellow are also active site residues and are shared between the two binding pockets.

In Figure 2, the active site residues together with all the positions subjected to mutagenesis and the substitutions selected for each position can be seen.

### 3.2. Specific Activities with Alternative Substrates

The impact of the structural changes to each mutant caused by the different substitutions was probed by determining the specific activities with four alternative substrates (Figure 1) (Table 1, Table 2 and Table 3). Each substrate represents a distinct reaction type commonly catalyzed by GSTs. CDNB undergoes nucleophilic aromatic substitution (Figure 1) and is considered a universal substrate for GSTs. Isothiocyanates such as PEITC are naturally occurring compounds in many cruciferous vegetables such as broccoli [31], and PEITC can undergo nucleophilic addition (Figure 1) to form a dithiocarbamate [32,33]. EA is a diuretic drug that can serve both as a substrate and an inhibitor of GSTs [34,35], and EA can undergo a Michael addition catalyzed by GSTs (Figure 1) [36]. CuOOH is a synthetic substrate for GSTs, which represents the endogenous products of lipid peroxidation. CuOOH can undergo hydroperoxide reduction catalyzed by GSTs (Figure 1) [37].

The reaction monitored by CuOOH differs from the other substrates in that it is not a standard conjugation with only GSH but rather a three-component coupled reaction including NADPH and glutathione reductase. Unlike the other reactions where the formation of product is being monitored, it is instead the consumption of NADPH being assayed. In the reaction mechanism activated GSH first makes a nucleophilic attack on the oxygen of the hydroxyl group of CuOOH to release an alcohol (1).

(1) CuOOH+GSH→CuOH+GSOH

From here, the unstable sulfenic acid of glutathione, GSOH, can react with another molecule of GSH to form glutathione disulfide (GSSG) and water (2).

(2) $$GSOH+GSH\to GSSG+H_{2}O$$

Lastly, the reduced form of glutathione is regenerated from the disulfide by the aid of NADPH together with glutathione reductase (3).

(3) $$GSSG+NADPH+H^{+}\to 2\;GSH+NADP^{+}$$

**Figure 3:**
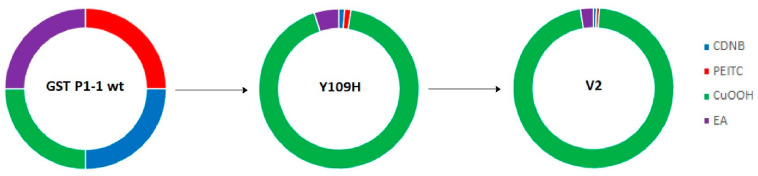
Circular diagrams illustrating the change in substrate selectivity profiles of Y109H and V2 with four alternative substrates relative to wildtype human GST P1-1. The specific activities of human wildtype GST P1-1 have been given the same weight with each substrate. The colored segments of Y109H and V2 (Q40M-E41Q-A46S-Y109H-V200L) represent the fractional activity with each substrate relative to wildtype GST P1-1.

The most noteworthy change in GST P1-1 activities following mutations was obtained with the substrate CuOOH (Figure 3). The substrate selectivity profiles of enzymes acting on alternative substrates can aptly be illustrated in circular (doughnut) diagrams [38]. The activities of wildtype GST P1-1 are used as standards for the comparison of the enzymes such that all substrates are given the same contribution to the plot. The activities of mutant GSTs are then calculated as the fraction of GST P1-1 activity for each one of the alternative substrates. The different graphs show the fractional activities of the sum of the total activities with the alternative substrates obtained for a given enzyme [13].

The single-point mutant R1 (Y109H) was shown to have a 16.3-fold increase in CuOOH activity, while decreased activity was observed with CDNB (5.1-fold), PEITC (4.5-fold), and EA (1.15-fold) compared to wildtype GST P1-1 (Table 4, Figure 3). Further improvement in CuOOH substrate selectivity was noted with the mutants V1 (T35S-Q40L-A46S-Q85R-Y109H), V2 (Q40M-E41Q-A46S-Y109H-V200L), V3 (Q40L-S43P-Q85K-Y109H-V200L), as well as with V8 (Q40L-E41Q-Q84P-Y109H-V200A), among which the most prominent was V2 (Table 4). The CuOOH activity of V2 was 30-fold higher than that of wildtype GST P1-1, while the activities with the other substrates were in the same range as observed with Y109H (Table 4, Figure 3).

The mutant R211 (Y109H-V36K) showing highest activity with CDNB and PEITC displayed a 3.9-, 3-, and 1.3-fold lower activity with CDNB, PEITC, and EA, respectively, while the CuOOH activity was increased 3.6-fold compared to the wildtype enzyme (Table 4). The only mutant to show increased EA activity (1.2-fold) compared to wildtype GST P1-1 was V8 (Q40L-E41Q-Q84P-Y109H-V200A) (Table 4). Additionally, the activity with CuOOH was 23-fold higher than that of wildtype GST P1-1 (Table 4).

The V3XX mutants (Table 3) were likewise tested with all four substrates (Figure 1) in order to evaluate their catalytic competence, and several of the mutants displayed no catalytic function with one or two alternative substrates, although minor activity was present with the other substrates (Table 3). For example, the mutants V304 (E41Q-Q84P-Q85K-S106T-Y109H-S185C-G206E), V311 (E41Q-Q84P-Q85K-I105D-S106T-Y109H-S185C-G206D), V312 (E41Q-Q84P-Q85K-I105E-S106T-Y109H-S185C-G206E), and V313 (E41Q-Q84P-Q85K-I105E-S106T-Y109H-S185C-S185C-G206D) had no measurable activity with CuOOH (Table 3), while V314 (P10H-E41Q-Q84P-Q85K-I105D-S106T-Y109H-S185C-G206E), V316 (P10H-E41Q-Q84P-Q85K-I105E-S106T-Y109H-S185C-G206E), and V317 (P10H-E41Q-Q84P-Q85K-I105E-S106T-Y109H-S185C-G206D) had no measurable activity with CuOOH or PEITC (Table 3). The mutant V315 (P10H-E41Q-Q84P-Q85K-I105D-S106T-Y109H-S185C-G206D) was found to be non-functional with all substrates except for a weak activity with CDNB (Table 3). As mentioned, the V3XX mutants were based on the V205 mutant (E41Q-Q84P-Q85K-S106T-Y109H-S185C), and in general, the increased number of mutations added decreased the functionality of the enzyme. Furthermore, G206D was the least deleterious mutation, followed by P10H, and the most unfavorable mutation was I105E. Moreover, glutamic acid seemed to have a more negative impact on the activity than aspartic acid (Table 3). Apart from the V3XX mutants, poor catalytic ability was also identified with the single-point mutants Y8E/H, and the double-point mutants Y109H-V11E/H/S as well as Y109H-F9H (Table 1).

### 3.3. Multivariate Data Analysis of Activity Profiles

**Figure 4:**
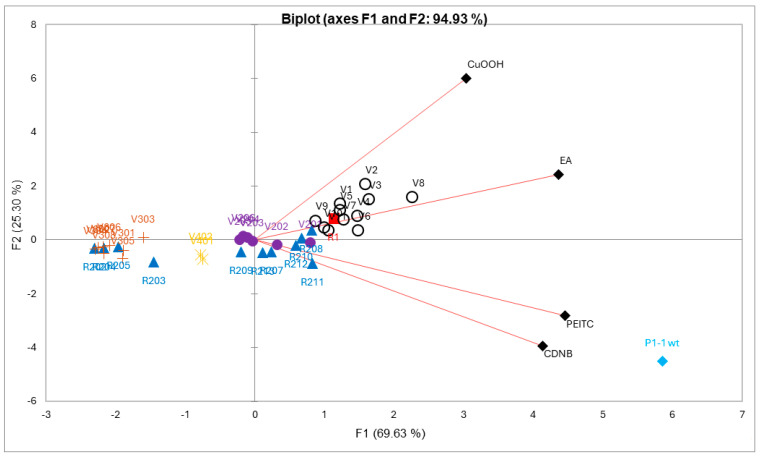
A principal component analysis of mutant library data with respect to enzyme activities acting on alternative substrates. The biplot is a representation of the scores (activities of enzymes) and loadings (substrates) in the 2D plane defined by PC1 and PC2. The activities were standardized prior to analysis to fit the same scale. Each enzyme has been color-coded according to their respective mutant library and represents a four-dimensional vector based on measured variables (CDNB, PEITC, CuOOH, and EA). Scores that are close to each other show similar substrate selectivity, and scores located closer to loadings show higher selectivity for that substrate.

For the multivariate analysis, all mutants with incomplete data points in one or more dimensions, due to unmeasurable experimental data, were removed. For the remaining 40 mutants, data points corresponding to specific activities (µmol^−1^ min^−1^ mg^−1^) were analyzed. A PCA plot was constructed to evaluate the difference between the mutant enzymes with respect to catalytic performance with the four alternative substrates (Figure 4). The obtained loadings (variables) and scores (observation) can be combined into a biplot, illustrating which loadings have the largest effect on each component and how the loadings relate to one another as well as how the scores relate to the loadings. In the biplot, Figure 4, each mutant represents a four-dimensional vector in the functional space, the first principal component (PC1) and the second principal component (PC2) captured a combined 94.93% of the total variation in the data, with 69.63% and 25.30% captured by PC1 and PC2, respectively. PC1 is influenced by all variables. PC2 is more influenced by CuOOH and to a lesser extent by EA, and negatively influenced by PEITC, and CDNB. Moreover, CDNB and PEITC appear to be positively correlated, while CDNB and CuOOH are not correlated.

Wildtype GST P1-1 has a clear distinction in terms of substrate selectivity towards CDNB and PEITC compared to the other groups (Figure 4). R1 (Y109H), red score, is a single-point mutant with a preference for CuOOH and EA (Figure 4). Interestingly, the variants belonging to the VX mutant library (black circle scores) show further improved selectivity in the CuOOH and EA direction. A closer examination of the more prominent mutants with this newly gained function, V1 (T35S-Q40L-A46S-Q85R-Y109H), V2 (Q40M-E41Q-A46S-Y109H-V200L), V3 (Q40L-S43P-Q85K-Y109H-V200L), and V8 (Q40L-E41Q-Q84P-Y109H-V200A), all share a common feature, alterations to position 40, although in the presence of other substitutions. V1, V3, and V8 have Q40L, while V2, which displayed the highest preference for CuOOH, has Q40M. Furthermore, V8 also showed the strongest preference for EA out of all tested mutants (Table 2) and compared to the second most active mutant, V9 (T35S-S43P-C102S-Y109H-V200A), they both share V200A. The R2XX library (blue scores) and V20X library (purple scores) were mostly scattered around the center (Figure 4), meaning that these mutants did not show selectivity towards any particular substrate compared to other mutants, with a few exceptions located along the CDNB and PEITC loadings, the most active being R211 (Y109H-V36K) and R212 (Y109H-V36I), both containing changes to position 36. The strong preference of wildtype GST P1-1 for CDNB and PEITC overshadows other mutants, making them less apparent along the same loadings. However, looking at a PCA plot without the wildtype enzyme, it is clear that V6 (black circle score, Q85R-C102S-S106T-Y109H-V200L) is located closer to the CDNB and PEITC loadings and show higher CDNB activity compared to the other VX mutants. Although the reason is not fully clear, the mutant V401 (Q85R-Y109H) and V403 (Q85R-Y109H-V200L) give a hint that the substitution C102S and/or S106T may not only be responsible for the improved CDNB activity but also play a role in PEITC activity (Table 2).

The V3XX mutants (brown scores) were located in the far left-hand side of the biplot, opposite of the loadings, together with part of the R2XX library mutants (blue scores), meaning that these mutants had an overall poor activity with all substrates. The V3XX mutants were based on the mutant V205, and adding either P10H, I105D/E, or G206D/E as in V301-V305, respectively, caused a severe reduction in activity with all substrates. The V40X mutants were located in between the V3XX and V2X mutants (purple scores), but closer to the V20X mutants, and comparing the mutant V401 (Q85R-Y109H) to V403 (Q85R-Y109H-V200L), the added Leu in position 200 does not appear to have any significant effect.

### 3.4. Design of a New Set of Mutants for the Identification of Substitutions Relevant to CuOOH Activity

The most frequently appearing substitutions among mutants with the highest CuOOH activivity (V1, V2, V3, and V8, Table 4) were selected to construct new double-point mutants to identify substitutions responsible for the enhanced CuOOH activity beyond that of the Y109H mutant (Table 4). In addition, the effects from Q85R and Q85R in conjunction with V200L could also be evaluated from the V401 (Q85R-Y109H) and V403 (Q85R-Y109H-V200L) mutants available from previous mutant libraries. The largest effect on the CuOOH activity was observed with V501 (Q40M-Y109H), V502 (Q40L-Y109H), and V503 (E41Q-Y109H). In these the increase was 1.2-, 1.4-, and 1.3-fold, respectively, compared to the Y109H mutant (Table 5), signifying that these substitutions play a role in CuOOH activity. The other substitutions appeared to have a rather neutral effect on the activity (Table 5).

However, none of the individual activities for the double-point mutants matches 0.9 μmol min^−1^ mg^−1^ measured for the most active mutant, V2 (Q40M-E41Q-A46S-Y109H-V200L).

### 3.5. Determination of Steady-State Kinetic Parameters with CuOOH

The change in the steady-state kinetic parameters following the evolutionary path of the wildtype enzyme to the most CuOOH-active enzyme was investigated by using CuOOH as the substrate. We first established that the coupling reaction between NADPH and GSSG reductase was not limited by the concentration of GSSG reductase at the highest concentration of CuOOH; 0.3 unit/mL of GSSG reductase was enough even at the highest concentration of CuOOH used in the assay. It should also be noted that the 1 mM glutathione used is in the physiological range, which essentially is a concentration saturating GST P1-1.

The k_cat_ values of the mutants Y109H and V2 are 34- and 57-fold higher than that of the wildtype enzyme (Table 6). The K_m_ values of Y109H and V2 are similar and approximately 3-fold higher than the value of the wildtype enzyme. The resulting catalytic efficiencies (k_cat_/K_m_) for Y109H and V2 are 10- and 23-fold higher than the value for wildtype GST P1-1, respectively. In summary, the increased activity with CuOOH noted in the mutants is primarily caused by elevated k_cat_ values.

## 4. Discussion

### 4.1. Physiological Role of Hydroperoxide Reduction

Aerobic organisms are challenged by diverse reactive molecules formed by the reduction of molecular oxygen, and their tissues are protected by a variety of enzymes [40]. Among the toxicants are organic hydroperoxides (ROOH), which can be detoxified by reduction to corresponding alcohols (ROH) and water in reactions catalyzed by glutathione peroxidase.

(4) $$ROOH+2\;GSH\to ROH+H_{2}O+GSSG$$

The organic hydroperoxides formed physiologically include numerous fatty acid derivatives, steroids, and oxidation products of nucleic acids. As surrogates for these substrates the chemically more stable cumene hydroperoxide (CuOOH) has been found useful for enzyme characterization. Two major categories of glutathione peroxidase have been identified as follows [7]: selenocysteine-containing proteins [41] and non-selenium-dependent enzymes in the form of GSTs [42], and both types play significant protective roles. Among the mammalian cytosolic GSTs, the alpha class members are in general the most efficient peroxidases with specific activities >1 µmol min^−1^ mg^−1^ [43], whereas wildtype GST P1-1 displays significantly lower specific activity with CuOOH Table 4). The mutational analysis shows that a single-point mutation can raise the hydroperoxidase activity from 0.03 µmol min^−1^ mg^−1^ to 0.49 µmol min^−1^ mg^−1^, and that a second mutation can further elevate the value up to 0.70 µmol min^−1^ mg^−1^ (Table 5). From the evolutionary perspective it should be noted that the discrimination between alternative substrates is markedly altered by the mutations. The activity of mutant V2 with PEITC is reduced 5-fold while the activity with CuOOH is increased 30-fold (Table 4) corresponding to a change in the relative activities of 150-fold. The substrates PEITC and CuOOH represent physiologically relevant reactions, and evolutionary scenarios can be envisioned in which enhancing one process while suppressing another provides a selective advantage. Enzymes acting on alternative substrate acceptance have to navigate a multidimensional fitness landscape in evolution [44].

### 4.2. Structure–Activity Relationship

**Figure 5:**
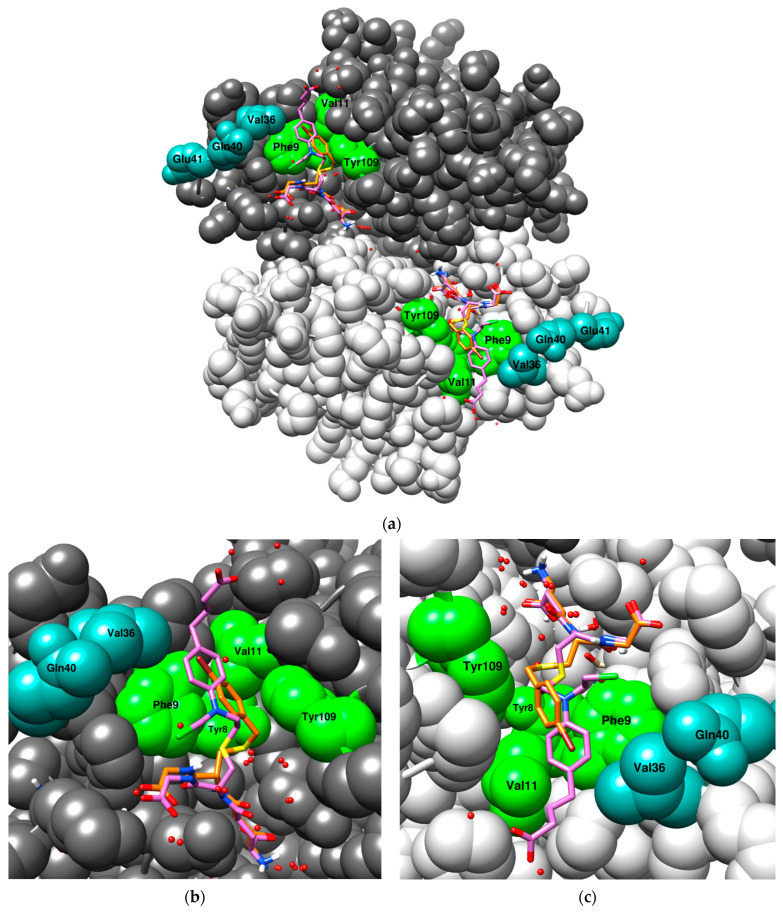
The superposition of two crystal structures of human GST P1-1 with their respective co-crystallized ligand: S-(p-bromobenzyl)-glutathione (1AQV, orange) and chlorambucil-GSH (3CSH, purple). A few of the residues interacting with the S-substituents are highlighted; green residues belong to the active site and teal residues belong to the α2-loop (Gln40 and Glu41 do not interact). (**a**) An overview of the full dimer with the ligands bound to the active site of each subunit; (**b**) a close-up of the first subunit, emphasizing the orientation of the GSH moiety leading up to the S-substituent; (**c**) a close-up of the second subunit, emphasizing the orientation of the S-substituents of both ligands. The model was generated in USCF Chimera (version 1.18) [52] with the program MODELLER [53].

A complete list of all incorporated substitutions together with the active site residues of GST P1-1 can be found in Figure 2. Amongst the active site residues, the constructed mutants contained modifications to H-site residues Phe9, Val11, Ile105, Gly206, as well as Tyr8 (which contributes to both H- and G-sites) (Figure 2 and Figure 5). In addition, Val36, Gln40, and Glu41 adjacent to the H-site were targeted. The substitutions that were observed to positively influence the CuOOH activity include Y109H, Q40M/L, and E41Q (Table 1 and Table 2), whereas the substitutions that were found to have a negative impact comprise Y8E/H, F9H, V11E/H/A/S/T, V36R/G/L/K/I/T (Table 1), P10H, I105E/D, as well as G206E/D (Table 3). These substitutions also had an overall negative impact on the other tested alternative substrates (Table 1 and Table 3).

Prior research has demonstrated that a point mutation, Y109V, in human GST P1-1 increased the activity with CuOOH 10-fold, while also decreasing the EA activity roughly 55-fold compared to the wildtype enzyme [11]. The same investigation also concluded that the actual contribution of Tyr109 as a catalytically active residue is dependent on the electrophilic substrate [10]. Moreover, crystallographic studies have indicated that the detrimental effect of Val109 on the EA activity is due to the absence of hydrogen bonding capability, in contrast to Tyr109, to stabilize the transition state leading to product formation [10,45]. Interestingly, our substitution in the same position, Y109H, did not only result in a 16.3-fold increase in CuOOH activity, improving the activity even further, but it also left the EA activity almost unaffected. Thus, the substrate selectivity can be tuned in evolution to fit an optimal trajectory in substrate-activity space. Histidine, which is another frequent active site residue with hydrogen bonding properties [46], apparently functions as a substitute for tyrosine with respect to the EA activity. The physical nature of histidine and valine are vastly different even though both residues enhance the CuOOH activity. Although it is not clear how, the increase in activity could possibly be ascribed to changes to the physical parameters such as hydrophobicity or volume. This can alter the orientation of the substrate and thereby the interactions, which in turn, can have pronounced effects on the activity [47].

To date there is no solved crystal structure of GST P1-1 in complex with hydroperoxide substrates such as CuOOH. The superposition of two human GST P1-1 crystal structures, one in complex with S-(p-bromobenzyl)-glutathione (1AQV, orange), and the other in complex with a GSH–chlorambucil conjugate (3CSH, purple), are shown in Figure 5a. Most of the N-terminus of the glutathione molecule is well aligned between the two structures, with slight deviations at the C-terminus. However, the largest deviation for the glutathione molecule occurs close to the thioether group (Figure 5b, sulfur atom in yellow). Comparing the two structures beyond the S-substituent, the aromatic ring of chlorambucil overlaps with the bromine atom of p-bromobenzyl; however, the aromatic ring adopts a slightly different conformation and does not perfectly align with the outline of the aromatic ring of p-bromobenzyl. Moreover, it can form π-electron interactions with Phe9, as well as van der Waals interactions with Val11 and Val36, amongst other residues [48] (Figure 5c), whereas the aromatic ring of p-bromobenzyl is sandwiched between the aromatic rings of Tyr109 and Phe9 (Figure 5c) [49]. Structurally, CuOOH (Figure 1) is more similar to chlorambucil, viewed from the sulfur of GSH up to the aromatic ring, making it more likely that CuOOH adopts a similar orientation as chlorambucil in the H-site. Examining the effect of changes to Val36 (Table 1) it is evident that hydrophobic residues in this position are more advantageous than non-hydrophobic residues with respect to the CuOOH activity, supporting the notion of ongoing hydrophobic interactions between Val36 and the aromatic ring or perhaps the methyl groups of CuOOH. Furthermore, the mutant R203 (V11A-Y109H) showed a 10-fold decrease in CuOOH activity compared to the Y109H mutant. Ala is the most neutral of all amino acids, and it can be used to probe the contribution of a specific residue [50,51]. This suggests that the H-site residue Val11 forms important hydrophobic interactions with CuOOH, possibly like the ones in chlorambucil.

Epistatic effects can be described as non-additive effects between mutations, meaning that the contribution of two or more substitutions exceeds the expected effect of the individual substitutions added together. Moreover, epistatic effects are more common among activity improving substitutions as well as residues more tolerant to substitutions and can significantly change the effect of substitutions [54,55,56,57,58,59]. Further improvement in CuOOH activity could be observed with the second generation VX mutant library; these mutants form a large cluster surrounding the R1 (Y109H) mutant in the PCA-plot (Figure 4), with the most active mutants along the CuOOH trajectory being V1 (T35S-Q40L-A46S-Q85R-Y109H), V2 (Q40M-E41Q-A46S-Y109H-V200L), V3 (Q40L-S43P-Q85K-Y109H-V200L), and V8 (Q40L-E41Q-Q84P-Y109H-V200A). The substitutions present in these mutants promote activity towards CuOOH, whereas they did not significantly alter the activity with the other substrates. In examining the crystal structure of human GST P1-1 (Figure 5), many of the investigated residues are found near the N-terminal of the protein, more precisely, at the second alpha-helix (Figure 5, residues 35–46, highlighted in teal). This helix lies adjacent to the active site and forms part of the GSH binding pocket, and residues within this domain can have conformational flexibility to possibly facilitate binding and catalysis [27,28,60]. V2, the Y109H-based mutant with the highest CuOOH activity, had a 30-fold higher specific activity than the wildtype enzyme and an almost 2-fold elevated value compared to Y109H alone. The substitutions forming this mutant were individually examined by constructing double-point mutants (involving Y109H as one of the substitutions); 1.2- and 1.3-fold elevated activity was found with V501 (Y109H-Q40M) and V503 (Y109H-E41Q), respectively, while the other mutants had a rather neutral effect. However, this is almost 40% less than the CuOOH activity measured for V2, suggesting that epistatic interactions are operating among the substitutions in V2.

**Figure 6:**
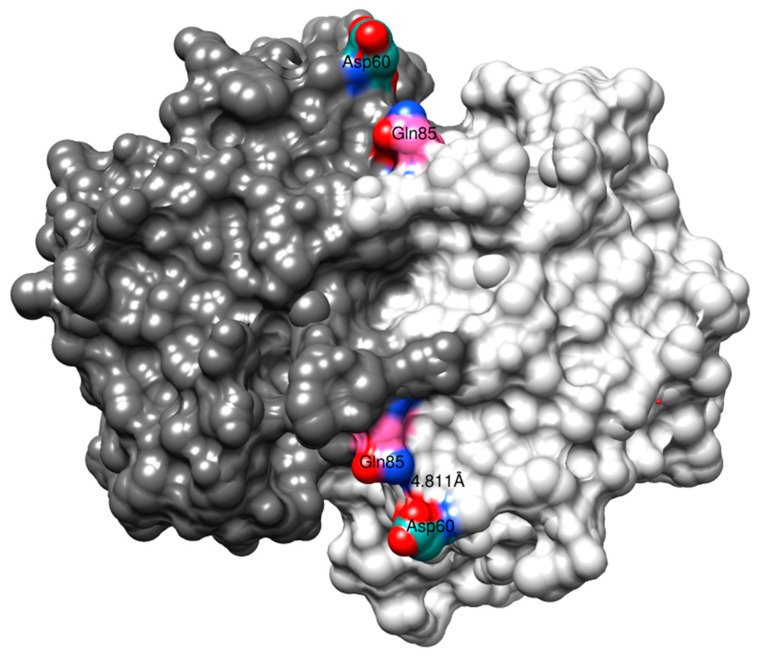
The crystal structure (1PGT) of human GST P1-1. The full dimer is displayed together with the possible Gln85–Asp60 interaction.

The substitutions outside the α2-helix, Q85R and V200L, did not significantly contribute to the CuOOH activity. Examining the 3D structure of human GST P1-1 with both subunits at display demonstrates that Gln85 is situated at the surface of the α4-helix, facing the opposing subunit, and has the ability to form a subunit–subunit interaction with Asp60 (Figure 6). Replacing Gln85 with Arg by modeling (USCF, Chimera) shows that the sidechain guanidinium group has the ability to form additional hydrogen bonding with the sidechain carboxamide group of Asp60 (not shown). However, Arg85–Asp60 inter-subunit interactions did not appear to have any influence on the activity of the enzyme (Table 2), even though previous research has suggested that this particular interaction positively contributes to increased thermostability of the enzyme [22].

The most effective double-point mutant with respect to the CuOOH activity was V502 (Q40L-Y109H) with 1.4-fold enhanced activity compared to Y109H. This particular substitution was present in the other highly CuOOH-active mutants V1, V2, and V8. Neither Met or Leu possess catalytic properties, and modeling (UCSF Chimera) showed that both residues in position 40 flank the active site, just as the native residue, and could possibly influence the peroxidase activity through hydrophobic interaction with the substrate molecule. Moreover, none of the mutated residues connected to the α2-loop formed inter-subunit interactions (Figure 5a).

A handful of substitutions also impaired the overall catalytic competence of the enzyme with the tested substrates, including the CuOOH activity. The most detrimental ones were found in the active site of the enzyme (Figure 5a, green residues). Tyr8 is an evolutionarily conserved active site residue, which is essential for efficient GSH activation in many classes of cytosolic GSTs [61], and neither of the substitutions in R2 (Y8H) or R3 (Y8E) could serve as a substitute (Table 1). The activity was completely abolished in R2, while R3 had only 0.08% remaining activity with CDNB. Phe9 (Figure 5a, green residue) is one of eight residues forming the H-site, and the mutant R206 (F9H-Y109H) also resulted in severe activity loss with nullified CuOOH activity and only trace amounts of CDNB, PEITC, and EA activity (Table 1). Phe9 forms the side walls of the H-site together with Tyr109 [49] and is involved in hydrophobic contacts with the electrophilic substrate as well as with the C-terminal carbon-chain of GSH [62], whereas the more polar histidine cannot form such interactions.

Moving across the generations, from the VX to V2XX to V3XX mutant libraries, appeared to cause a gradual loss in the overall activity (Table 2 and Table 3). The observed effect in these cases is most likely linked to diminishing returns in activity as the number of added substitutions increases [63,64]. For example, the H-site residue 105 is naturally polymorphic and occurs most commonly as either Ile or Val, and the Val variant has been shown to be less active with a number of electrophilic substrates [39]. Adding I105D/E to the V205 mutant as in V302/3 almost nullified the activity with all four alternative substrates (Table 3). Similarly, adding G206D/E to the V205 mutant as in V304/5 had a detrimental effect on the activity. The addition of G206E caused a complete loss of CuOOH activity, with only fractional activity remaining for the other substrates (Table 3). Interestingly, G206D still retained almost 50% of the CDNB and PEITC activities, while a complete absence of CuOOH activity and only traces of EA activity were observed (Table 3). The 3D structure of human GST P1-1 reveals that Gly206 (H-site residue) forms the bottom part of the H-site pocket together with Val11 [49], and that the backbone nitrogen of Gly206 can form a hydrogen bond with the hydroxyl group of Tyr109 in the presence of bound GSH. Moreover, the introduced substitutions could also possibly evoke negative epistasis, an effect that has been observed in alpha class GSTs and other enzymes [44,65].

To what extent the evolved CuOOH activity extends to other more physiologically relevant organic hydroperoxides remains to be investigated. Our preliminary results have shown that the mutant Y109H has a 3-fold higher activity with t-butyl hydroperoxide compared to the wildtype enzyme. Judging from the available data, GST activity with one organic hydroperoxide is commonly accompanied by activity with analogous compounds, suggesting enhanced functionality with various substrates [66].

## 5. Conclusions

The single-point active site mutation Y109H in GST P1-1 was found to increase the CuOOH activity 16.3-fold. This enhanced peroxidase function was obtained without causing any major changes to the EA activity of the wildtype enzyme. The effect was primarily effected through a major increase in k_cat_. The mutant V2 (Q40M-E41Q-A46S-Y109H-V200L), based on Y109H, further elevated the CuOOH activity almost 2-fold compared to Y109H by an additional increase in k_cat_. The substrate selectivity of an enzyme is governed by k_cat_/K_m_ and this parameter has therefore been referred to as the specificity constant [67,68]. Toxic substrates such as hydroperoxides are normally produced in low concentrations under physiological conditions and the rate of the detoxication reaction will then primarily be determined by k_cat_/K_m_. The present investigation shows that a small number of mutations can significantly alter the preference of an enzyme for one substrate over others. This can play an important role in an evolutionary context where one function could diverge into another function in response to selection pressure. On the other hand, it may also be an advantage in the course of evolution if a promiscuous enzyme could selectively enhance the catalysis of a particular reaction while maintaining activity with alternative substrates. Our results suggest that different combinations of mutations may accomplish one or the other of these alternatives.

## Figures and Tables

**Table 1:** Specific activities (µmol^−1^ min^−1^ mg^−1^) of wildtype human GST P1-1 and the GST P1-1 mutants from the RX and R2XX libraries with four alternative substrates. ND = Not detectable.

	*Specific Activity* *(μmol min*^−1^ *mg*^−1^*)* Substrate			
GST P1-1 Variant	CDNB	PEITC	CuOOH	EA
P1-1	106 ± 4	60 ± 3.0	0.030 ± 0.001	2.00 ± 0.10
R1 (Y109H)	20.9 ± 0.7	13.4 ± 0.1	0.49 ± 0.03	1.7 ± 0.1
R2 (Y8H)	0.080 ± 0.004	ND	ND	ND
R3 (Y8E)	ND	ND	ND	ND
R201 (Y109H-V11E)	0.027 ± 0.002	ND	ND	0.047 ± 0.004
R202 (Y109H-V11H)	0.0097 ± 0.0008	0.85 ± 0.07	0.03 ± 0.01	0.031 ± 0.001
R203 (Y109H-V11A)	17.5 ± 0.4	4.31 ± 0.06	0.05 ± 0.01	0.14 ± 0.01
R204 (Y109H-V11S)	1.82 ± 0.02	0.46 ± 0.02	0.024 ± 0.004	0.16 ± 0.01
R205 (Y109H-V11T)	5.2 ± 0.2	0.31 ± 0.03	0.066 ± 0.004	0.21 ± 0.01
R206 (Y109H-F9H)	0.71 ± 0.01	0.42 ± 0.01	ND	0.046 ± 0.007
R207 (Y109H-V36R)	20.6 ± 1.2	11.9 ± 1.6	0.09 ± 0.01	1.46 ± 0.06
R208 (Y109H-V36M)	24.4 ± 1.1	13.3 ± 0.8	0.44 ± 0.03	1.29 ± 0.02
R209 (Y109H-V36G)	12.4 ± 0.3	14.5 ± 0.5	0.10 ± 0.02	1.06 ± 0.08
R210 (Y109H-V36L)	22.0 ± 0.3	15.3 ± 0.8	0.35 ± 0.02	1.24 ± 0.04
R211 (Y109H-V36K)	27.0 ± 2.5	19.8 ± 0.8	0.11 ± 0.01	1.38 ± 0.06
R212 (Y109H-V36I)	18.5 ± 0.2	18.8 ± 0.7	0.27 ± 0.09	1.19 ± 0.09
R213 (Y109H-V36T)	15.6 ± 0.2	15.5 ± 0.2	0.11 ± 0.02	1.23 ± 0.07

**Table 2:** The specific activities (µmol^−1^ min^−1^ mg^−1^) of wildtype human GST P1-1 and the GST P1-1 mutants from the VX, V20X, V40X, and V50X libraries with four alternative substrates. NM = Not measured.

	*Specific Activity* *(μmol min*^−1^ *mg*^−1^*)* Substrate			
GST P1-1 Variant	CDNB	PEITC	CuOOH	EA
P1-1	106 ± 4	60 ± 3.0	0.030 ± 0.001	2.00 ± 0.10
V1 (T35S-Q40L-A46S-Q85R-Y109H)	20.5 ± 1.2	12.5 ± 0.2	0.70 ± 0.10	1.50 ± 0.10
V2 (Q40M-E41Q-A46S-Y109H-V200L)	19.2 ± 0.7	11.8 ± 0.5	0.90 ± 0.20	1.66 ± 0.08
V3 (Q40L-S43P-Q85K-Y109H-V200L)	20.6 ± 0.5	16.8 ± 1.8	0.80 ± 0.20	1.53 ± 0.06
V4 (T35S-E41Q-Q85K-S106T-Y109H)	20.7 ± 0.6	18.7 ± 0.4	0.61 ± 0.05	1.55 ± 0.02
V5 (Q40M-S43P-Q85R-Y109H-S185C)	17.5 ± 0.5	14.4 ± 0.4	0.58 ± 0.06	1.70 ± 0.10
V6 (Q85R-C102S-S106T-Y109H-V200L)	28.2 ± 0.3	15.6 ± 0.5	0.43 ± 0.08	1.80 ± 0.10
V7 (A46S-S106T-Y109H-S185C-V200A)	19.7 ± 0.2	16.0 ± 1.6	0.50 ± 0.10	1.72 ± 0.03
V8 (Q40L-E41Q-Q84P-Y109H-V200A)	21.3 ± 1.3	17.0 ± 1.4	0.70 ± 0.10	2.40 ± 0.10
V9 (T35S-S43P-C102S-Y109H-V200A)	17.9 ± 1.1	10.0 ± 0.6	0.36 ± 0.02	1.94 ± 0.08
V10 (Q40M-Q84P-Q85K-C102S-Y109H)	20.3 ± 0.4	15.9 ± 0.3	0.44 ± 0.06	1.48 ± 0.05
V11 (T35S-Q84P-Y109H-S185C-V200L)	21.9 ± 0.3	17.2 ± 0.8	0.45 ± 0.06	1.39 ± 0.06
V201 (T35S-Q40L-E41Q-Q84P-Q85K-S106T-Y109H)	22.4 ± 1.1	18.6 ± 3.6	0.35 ± 0.02	1.17 ± 0.21
V202 (T35S-Q40L-E41Q-Q85K-S106T-Y109H-S185C)	22.2 ± 1.3	13.4 ± 3.6	0.28 ± 0.02	1.06 ± 0.24
V203 (T35S-E41Q-Q84P-Q85K-S106T-Y109H-S185C)	14.0 ± 0.2	12.8 ± 2.1	0.23 ± 0.01	1.07 ± 0.03
V204 (T35S-Q40L-E41Q-Q84P-Q85K-S106T-Y109H-S185C)	14.1 ± 0.5	11.5 ± 0.4	0.28 ± 0.02	0.98 ± 0.04
V205 (E41Q-Q84P-Q85K-S106T-Y109H-S185C)	13.1 ± 1.0	9.68 ± 1.6	0.19 ± 0.01	1.16 ± 0.14
V206 (Q40L-E41Q-Q84P-Q85K-S106T-Y109H-S185C)	14.6 ± 0.4	9.06 ± 1.4	0.26 ± 0.02	1.08 ± 0.06
V401 (Q85R-Y109H)	20.2 ± 0.7	10.8 ± 0.3	0.54 ± 0.04	1.7 ± 0.011
V402 (Q85R-Y109H-V200L)	18.5 ± 0.4	11.2 ± 0.2	0.50 ± 0.08	1.84 ± 0.02
V501 (Q40M-Y109H)	NM	NM	0.59 ± 0.09	NM
V502 (Q40L-Y109H)	NM	NM	0.70 ± 0.12	NM
V503 (E41Q-Y109H)	NM	NM	0.63 ± 0.08	NM
V504 (S43P-Y109H)	NM	NM	0.53 ± 0.04	NM
V505 (A46S-Y109H)	NM	NM	0.50 ± 0.08	NM

**Table 3:** The specific activities (µmol^−1^ min^−1^ mg^−1^) of wildtype human GST P1-1 and the GST P1-1 mutants from the V3XX library with four alternative substrates. ND = Not detectable.

	*Specific Activity* *(μmol min*^−1^ *mg*^−1^*)* Substrate			
GST P1-1 Variant	CDNB	PEITC	CuOOH	EA
P1-1	106 ± 4	60 ± 3	0.030 ± 0.001	2.0 ± 0.1
V301 (P10H-E41Q-Q84P-Q85K-S106T-Y109H-S185C)	4.86 ± 0.02	2.52 ± 0.21	0.030 ± 0.001	0.23 ± 0.01
V302 (E41Q-Q84P-Q85K-I105D-S106T-Y109H-S185C)	0.130 ± 0.008	0.050 ± 0.006	0.011 ± 0.001	0.17 ± 0.01
V303 (E41Q-Q84P-Q85K-I105E-S106T-Y109H-S185C)	0.060 ± 0.005	0.09 ± 0.01	0.018 ± 0.001	0.092 ± 0.020
V304 (E41Q-Q84P-Q85K-S106T-Y109H-S185C-G206D)	7.89 ± 0.60	4.11 ± 0.40	ND	0.046 ± 0.002
V305 (E41Q-Q84P-Q85K-S106T-Y109H-S185C-G206E)	0.85 ± 0.06	0.41 ± 0.04	ND	0.064 ± 0.004
V306 (P10H-E41Q-Q84P-Q85K-I105D-S106T-Y109H-S185C)	0.090 ± 0.004	0.008 ± 0.008	0.0140 ± 0.0004	0.38 ± 0.01
V307 (P10H-E41Q-Q84P-Q85K-I105E-S106T-Y109H-S185C)	0.090 ± 0.001	0.100 ± 0.003	0.0100 ± 0.0007	0.27 ± 0.02
V308 (P10H-E41Q-Q84P-Q85K-S106T-Y109H-S185C-G206E)	4.44 ± 0.10	1.00 ± 0.13	0.0010 ± 0.0004	0.061 ± 0.002
V309 (P10H-E41Q-Q84P-Q85K-S106T-Y109H-S185C-G206D)	11.4 ± 0.3	1.96 ± 0.17	0.0019 ± 0.0002	0.038 ± 0.001
V310 (E41Q-Q84P-Q85K-I105D-S106T-Y109H-S185C-G206E)	0.45 ± 0.02	0.007 ± 0.001	0.0020 ± 0.0009	0.024 ± 0.003
V311 (E41Q-Q84P-Q85K-I105D-S106T-Y109H-S185C-G206D)	0.14 ± 0.01	0.008 ± 0.001	ND	0.0040 ± 0.0006
V312 (P10H-E41Q-Q84P-Q85K-I105D-S106T-Y109H-S185C-G206E)	0.130 ± 0.004	0.013 ± 0.019	ND	0.038 ± 0.002
V313 (E41Q-Q84P-Q85K-I105E-S106T-Y109H-S185C-S185C-G206D)	0.180 ± 0.008	0.013 ± 0.002	ND	0.020 ± 0.001
V314 (P10H-E41Q-Q84P-Q85K-I105D-S106T-Y109H-S185C-G206E)	0.35 ± 0.02	ND	ND	0.017 ± 0.003
V315 (P10H-E41Q-Q84P-Q85K-I105D-S106T-Y109H-S185C-G206D)	0.070 ± 0.001	ND	ND	ND
V316 (P10H-E41Q-Q84P-Q85K-I105E-S106T-Y109H-S185C-G206E)	0.090 ± 0.001	ND	ND	0.031 ± 0.002
V317 (P10H-E41Q-Q84P-Q85K-I105E-S106T-Y109H-S185C-G206D)	0.170 ± 0.005	ND	ND	0.023 ± 0.002

**Table 4:** The specific activities of wildtype human GST P1-1, Y109H, and three Y109H-based mutants with alternative substrates. The substrate selectivity profile of human GST P1-1 [34,39] is added for comparison.

	*Specific Activity* *μmol min*^−1^ *mg*^−1^ Substrate			
GST P1-1 Variant	CDNB	PEITC	CuOOH	EA
Human GST P1-1	106 ± 4	60 ± 4	0.030 ± 0.001	2.0 ± 0.1
R1 (Y109H)	20.9 ± 0.7	13.4 ± 0.1	0.49 ± 0.03	1.7 ± 0.1
R211 (Y109H-V36K)	27.0 ± 2.5	19.8 ± 0.8	0.11 ± 0.01	1.38 ± 0.06
V1 (T35S-Q40L-A46S-Q85R-Y109H)	20.5 ± 1.2	12.5 ± 0.2	0.7 ± 0.1	1.5 ± 0.1
V2 (Q40M-E41Q-A46S-Y109H-V200L)	19.2 ± 0.7	11.8 ± 0.5	0.9 ± 0.2	1.66 ± 0.08
V3 (Q40L-S43P-Q85K-Y109H-V200L)	20.6 ± 0.5	16.8 ± 1.8	0.8 ± 0.2	1.53 ± 0.06
V8 (Q40L-E41Q-Q84P-Y109H-V200A)	21.3 ± 1.3	17.0 ± 1.4	0.7 ± 0.1	2.4 ± 0.1

**Table 5:** The specific activities (µmol^−1^ min^−1^ mg^−1^) of double-mutant enzymes with the substrate CuOOH. Y109H has been added for comparison.

	*Specific Activity (μmol min*^−1^ *mg*^−1^*)*						
Y109H	Q40M-Y109H	Q40L-Y109H	E41Q-Y109H	S43P-Y109H	A46S-Y109H	Q85R-Y109H	Q85R-Y109H-V200L
0.49 ± 0.03	0.59 ± 0.09	0.70 ± 0.12	0.63 ± 0.08	0.53 ± 0.04	0.48 ± 0.08	0.54 ± 0.04	0.51 ± 0.08

**Table 6:** Steady-state kinetic parameters for wildtype GST P1-1 and two mutant enzymes displaying the highest activity with the substrate CuOOH. The concentration of CuOOH ranged from 0.09 to 1.5 mM, while the concentration of GSH was kept constant at 1.0 mM.

Kinetic Parameter	GST P1-1 Variant		
	Wildtype	Y109H	V2
k_cat_ (s^−1^)	0.039 ± 0.005	1.31 ± 0.39	2.21 ± 0.44
K_m_ (mM)	1.25 ± 0.25	4.3 ± 1.6	3.26 ± 0.89
k_cat_/K_m_ (mM^−1^ s^−1^)	0.031 ± 0.005	0.31 ± 0.10	0.68 ± 0.16

## Data Availability

The data reported in this article are available upon reasonable request from the corresponding author.
